# The Role of the Norway Rat, *Rattus norvegicus*, as a Reservoir of Zoonotic Helminth Species in the City of Barcelona (Spain)

**DOI:** 10.3390/ani15030298

**Published:** 2025-01-21

**Authors:** Màrius Vicent Fuentes, Pablo Puchades-Colera, Carla Gosálvez, Sandra Sáez-Durán, Maria Cholvi-Simó, Santiago Ruvira, Joan Sanxis-Furió, Jordi Pascual, Rubén Bueno-Marí, Sandra Franco, Víctor Peracho, Tomás Montalvo, María Trelis, Ángela L. Debenedetti, María Teresa Galán-Puchades

**Affiliations:** 1 Parasites and Health Research Group, Department of Pharmacy, Pharmaceutical Technology and Parasitology, Faculty of Pharmacy and Food Sciences, University of Valencia, 46100 Burjassot, Valencia, Spain; pablopc@iata.csic.es (P.P.-C.); carla.go.can@gmail.com (C.G.); sandra.saez@uv.es (S.S.-D.); cholvimaria@gmail.com (M.C.-S.); santi.ruv.her@gmail.com (S.R.); josanfu91@gmail.com (J.S.-F.); rbueno@lokimica.es (R.B.-M.); maria.trelis@uv.es (M.T.); aldebenedetti@gmail.com (Á.L.D.); mteresa.galan@uv.es (M.T.G.-P.); 2 Pest Surveillance and Control, Agència de Salut Pública de Barcelona (ASPB), 08023 Barcelona, Spain; jpascualsala@gmail.com (J.P.); sfranco@asp.cat (S.F.); vperacho@aspb.cat (V.P.); tmontal@asp.cat (T.M.); 3 Department of Research and Development, Laboratorios Lokímica, 46980 Paterna, Valencia, Spain; 4 CIBERESP Epidemiology and Public Health, 08023 Barcelona, Spain

**Keywords:** *Rattus norvegicus*, reservoir, helminths, zoonoses, Barcelona, Spain

## Abstract

Synanthropic rodents are involved in the transmission of several parasitic zoonoses to humans, such as helminth parasites. As part of a multidisciplinary study concerning the rodent population in Barcelona (Spain), 300 specimens of the Norway rat, *Rattus norvegicus*, were analyzed to elucidate their helminth community, mainly species with a zoonotic potential. The sample included 263 specimens from the sewage system and 37 from public gardens. A total of 253 (84.3%) rats were found to be parasitized, and 206 (68.7%) harbored zoonotic species. Adult rats were found to be more heavily parasitized with zoonotic helminths than juveniles, but the sex and site of capture had no influence. Six zoonotic helminths were identified: *Hydatigera taeniaeformis* larvae (1.7%), *Rodentolepis nana* (8.0%), *Hymenolepis diminuta* (21.3%), *Calodium hepaticum* (46.3%), *Gongylonema neoplasticum* (36.7%), and *Moniliformis moniliformis* (2.3%). Human zoonotic helminth infections often go unreported, so that the role of *R. norvegicus* in their transmission is unknown. According to our results, it is advisable to monitor and control rodent populations in deprived settlements close to cities and in cities themselves, and to promote good hygienic and sanitary practices, especially among vulnerable populations and workers with high exposure such as sewage workers.

## Simple Summary

Urban rat species, Norway and black rats, are involved in the transmission of several zoonotic pathogens to humans, such as helminth parasites. As part of a multidisciplinary study concerning the rodent population in Barcelona (Spain), 300 specimens of the Norway rat were analyzed to elucidate their role in the transmission of zoonotic helminths. The sample included 263 specimens from the sewage system and 37 from public gardens. A total of 84.3% of the rats analyzed were found parasitized, and 206 (68.7%) harbored zoonotic species. Adult rats were found to be more heavily parasitized with zoonotic helminths than juveniles, but sex and site of capture had no influence. Six zoonotic helminths were identified: *Hydatigera taeniaeformis* larvae, *Rodentolepis nana*, *Hymenolepis diminuta*, *Calodium hepaticum*, *Gongylonema neoplasticum*, and *Moniliformis moniliformis*. Human zoonotic helminth infections often go unreported, so the role of *R. norvegicus* in their transmission is unknown. According to our results, it is advisable to monitor and control rodent populations in deprived settlements close to cities and in cities themselves, and to promote good hygienic and sanitary practices, especially among vulnerable populations and workers with high exposure such as sewage workers.

## 1. Introduction

The World Health Organization (WHO), in collaboration with the Food and Agriculture Organization of the United Nations (FAO), the United Nations Environment Programme (UNEP), and the World Organization for Animal Health (WOAH), established the concept of One Health to promote multi-sector responses to food safety hazards, zoonotic risks and other threats to public health in the interaction between humans, animals and the environment, and provide guidance on how to reduce these risks (https://www.who.int/news-room/questions-and-answers/item/one-health, accessed on 2 September 2024).

Human beings coexist in a close relationship with various animals and this interrelation can be a source of diseases with a marked impact on public health and the social and economic well-being of the world population. These diseases, transmissible from animals to humans, either by direct contact or through contamination of water, food or the environment, are known as zoonoses. In humans, more than 1400 pathogens are known, of which approximately 900 species are zoonotic (>60%), and 80% of the latter have the capacity to affect different animal species [1]. Zoonoses have certain characteristics that favor their spread, complicate their control and eventual eradication, and have a severe impact on human and animal health [2].

Humans are affected by more than 100 parasitic zoonoses [3]. In urban areas, humans live with both synanthropic and domestic animals, usually small animals and pets, susceptible to the transmission of various protozoa and helminths, causing, almost always, non-occupational diseases and with a higher incidence in children. Among synanthropic animals, which take advantage of human habitats and food sources, rodents are the most common, together with insects, bats, pigeons and sparrows.

In recent decades, there has been an increase in diseases associated with small mammals that act as reservoirs [4]. Rodents are currently the most abundant and diverse order of mammals, representing 43% of the total number of mammalian species [5]. In peri-urban environments, rodents are a link between wild animals and humans, exposing the latter, even to zoonoses that usually tend to circulate in natural ecosystems. Rodents are reservoirs for a large number of infectious organisms that can be transmitted to humans and cause outbreaks of various zoonotic diseases with high morbidity and some mortality.

Synanthropic rodents of urban areas, *Rattus norvegicus* (Berkenhouth, 1769) (Norway rat) and *Rattus rattus* (Linnaeus, 1758) (black rat), act as active reservoirs (carriers and spreaders) of numerous pathogens (viruses, bacteria, and parasites) that can affect human health and therefore cause serious public health problems [6,7,8]. Human beings share cities with hundreds of thousands of rats and this contact between rats and humans can lead to the effective transmission of zoonotic diseases. Given the high rates of global urbanization [9], up-to-date knowledge of urban parasitic zoonoses is needed. It will thus be possible: (1) to evaluate the presence, magnitude, and nature of urban parasitic zoonoses in cities on a global scale; (2) to monitor and mitigate the emergency risk of urban parasitic zoonoses now and in the future; (3) to make health professionals aware of the presence and health risks of urban parasitic zoonoses and avert misdiagnosis of these diseases; and (4) to develop an effective rat population control strategy and other public health measures to reduce and prevent rat-human disease transmission [10]. The role of rodents as parasite reservoirs for protozoa and helminths is well known [4,11].

There are two transmission routes of zoonotic parasites from rodents in urban areas. In the first route of transmission, the rodent transmits the infective parasitic form directly to the human being, either cysts/oocysts, or eggs (for example, the protists *Giardia*, *Entamoeba*, *Blastocystis*, *Cryptosporidium*; the cestode *Rodentolepis nana* (Siebold, 1852), formerly *Hymenolepis nana*), which are, at the same time, the cause of self-infection of the rodent in monoxenous cycles in which the parasitic form is already infective. In the second route of transmission, the rodent acts as an indirect reservoir, since, in this case, it does not transmit the parasite directly to the human being, but rather reaches the human being, generally, via an arthropod, either by bite (such as the hemotissular protozoan *Leishmania*) or accidental ingestion of the same (for example, cockroaches or beetles in the cases of the tapeworm *Hymenolepis diminuta* (Rudolphi, 1819), the nematode *Gongylonema neoplasticum* (Fibiger et Ditlevsen, 1914) and the acanthocephalan *Moniliformis moniliformis* Bremser, 1811), or there are carriers, which are those hosts that directly transmit the parasite to humans. The carriers are both the predators of the rat as well as those that become infected with the parasitic forms emitted by the rat and are part of the human diet. In the first case, the carriers are the emitters of infectious forms for human beings, as in the case of the cat in the protozoan *Toxoplasma gondii* (Nicolle et Manceaux, 1909), or they become infected through the ingestion of the parasitized carrier which is part of the human diet, e.g., pigs in trichinosis (less important in urban settings) and snails in the case of angiostrongyliasis caused by *Angiostrongylus cantonensis* (Chen, 1935).

As Himsworth et al. [10] pointed out in their review, the role of rats as zoonotic parasite reservoirs has not been adequately analyzed in the most important cities of the world, with a few exceptions.

Concerning zoonotic parasitic helminths, in addition to *A. cantonensis*, detected in *R. norvegicus* practically at a cosmopolitan level, but also in *R. rattus*, other helminths were detected in urban rats in most of the studies carried out, and reported in the above-mentioned review: *Hymenolepis* spp. (*H. nana* and *H. diminuta*) and *Calodium hepaticum* (Bancroft, 1893) in *R. norvegicus*.

Regarding other zoonotic helminths in urban rats, various studies have been carried out in some European cities: Marseille (France) [12], Milan (Italy) [13], London (England) [14], Palermo (Italy) [15], Belgrade (Serbia) [16], Liverpool (England) [17], Hauts-de-Seine (France) [18], Helsinki (Finland) [19], and Budapest (Hungary) [20]; but also studies in cities in other countries around the word, such as Malaysia [21], Peru [22], Brazil [23], and South Africa [24].

In the case of Spain, studies of parasites in rodents of the genus *Rattus* have been carried out almost exclusively in rural and unpopulated areas, while studies in urban or peri-urban areas are scarce. Regarding helminthiases, these studies concern the cities of Granada [25], La Coruña and Santiago de Compostela [26] and Barcelona [27], and recently in Valencia [28], where, among other helminths and zoonotic parasites, *A. cantonensis* has been reported for the first time in continental Europe parasitizing *R. norvegicus* and *R. rattus* [29]. To sum up, the following zoonotic parasites have been reported in the urban sewer rat: *Brachylaima* sp. (Trematoda), *Hydatigera (=Taenia) taeniaeformis* (Batsch, 1786) larvae, *R. nana*, *H. diminuta* (Cestoda), *C. hepaticum*, *A. cantonensis*, *G. neoplasticum* (Nematoda) and *M. moniliformis* (Acanthocephala).

Previous results, published by our research group concerning the city of Barcelona [30], after the helminthological analysis of the first 100 rats in this city, showed the presence of 5 zoonotic helminth species, specifically the cestodes *R. nana* and *H. diminuta*, the nematodes *C. hepaticum* and *G. neoplasticum*, and the acanthocephalan *M. moniliformis*, which provides a glimpse into the spreading capacity and reservoir role of synanthropic rodents. These results should prompt public health authorities to address the situation within the concept of One Health, in which, disciplines such as public health in general, and the prevention of occupational risks in particular, must be involved.

Consequently, by updating the data on zoonotic helminth species in the Norway rat in the city of Barcelona, this research aims to shed light on the role *R. norvegicus* plays as a reservoir of zoonotic helminths, and warn the health authorities of parasites found in rats that can cause diseases in humans.

## 2. Materials and Methods

### 2.1. Study Area and Animals

To trap and dissect the rats, permission was granted by the Department of Territory and Sustainability of the regional government of Catalonia (reference number: SF/044), according to Directive 2010/63/EU of the European Parliament and Council decision of 22 September 2010 on the protection of animals used for scientific purposes. Then, to collect the rat individuals, snap traps were placed in the sewage system and rat cages in public gardens, always at night, when human access was restricted. All animals captured alive were euthanized by exposure to CO_2_-saturated atmosphere.

A total of 300 *R. norvegicus* caught from December 2016 to November 2017 at 35 different locations in the sewage system (n = 263) and in 10 different public gardens (n = 37) (Table 1), placed in populated residential neighborhoods, covering the 10 different districts of the city of Barcelona was helminthologically analyzed.

Rat individuals collected were dissected at the Pest Surveillance and Control Agency of Barcelona, and identified at a specific level, their sex determined, and weight and morphometry data were also collected and introduced in a database, together with data concerning the date and place of capture of each rat. Moreover, each rat was assigned to an age group according to their external morphometry, reproductive status and body weight as juveniles (<150 g) and adults (≥150 g) [31]. After dissection, the organs of each specimen were preserved in 95% ethanol. The helminthological analysis of preserved organs was carried out at the laboratory of the Parasites and Health Research Group of the Department of Parasitology of the University of Valencia.

### 2.2. Helminthological Procedures

All organs of each rat individual preserved in 95% ethanol, including the entire gastrointestinal tract, lungs, heart, pancreas, spleen, liver, kidneys and urinary bladder, were placed in Petri dishes containing saline and examined under a stereomicroscope. All helminths were collected and preserved in 70% ethanol. Then, all helminths were identified at a specific level based on their morphology and morphometry and according to the most relevant descriptions and findings of Cestoda [32,33,34], Nematoda [35,36,37,38,39,40,41] and Acanthocephala [42]. To do so, cestodes were stained with alcoholic hydrochloric carmine, differentiated with acidified ethanol, dehydrated in an alcohol series, cleared with xylene and mounted in Canada balsam, and nematodes were cleared in Amann lactophenol in non-permanent whole mounts.

### 2.3. Statistical Analysis

The number of parasitized hosts as well as the prevalence, mean abundance and range [43] of the helminth species found were analyzed.

Binary Logistic Regression (BRL) was applied to analyze the influence of the origin of capture, and the host sex and age on the prevalence of the zoonotic helminth species found. In the season-of-capture analyses, the four seasons were grouped in two groups, autumn–winter and spring–summer, due to the small number of captures, mainly during autumn and spring. Statistical significance was established at *p* < 0.05. The IBM SPSS Statistics 28.0 for Windows software package was used for statistical analysis.

## 3. Results

### 3.1. Helminth Species Found

The helminthological analysis of 300 *R. norvegicus* specimens from the city of Barcelona revealed that a total of 253 (84.3% [95% CI: 79.9–88.1]) rats were parasitized by at least one helminth species. Of these, 206 were carriers of at least one zoonotic helminth species (68.7% of the total rats analyzed [95% CI: 63.3–73.7]; 81.4% of the total parasitized rats [95% CI: 76.3–85.8]). The helminth species found (Table 2) were: 3 zoonotic cestodes, *H. taeniaeformis* larvae, *R. nana* and *H. diminuta*; 7 nematodes, 2 of them zoonotic, *C. hepaticum* and *G. neoplasticum*, and 5 non-zoonotic, *Eucoleus gastricus* (Baylis, 1926), *Aonchotheca annulosa* (Dujardin, 1843), *Trichosomoides crassicauda* (Bellinham, 1845), *Nippostrongylus brasiliensis* (Travassos, 1914) and *Heterakis spumosa* Schneider, 1866; and 1 zoonotic acanthocephalan, *M. moniliformis*. In addition, it should be noted that a total of 245 (81.7% [95% CI: 77.0–85.7]) rats were parasitized by helminth species with a monoxenous cycle (direct cycle, without intervention of intermediate hosts), and 94 rats (31.3% [95% CI: 26.3–36.7]) carried monoxenous helminth species only; furthermore a total of 159 (53.0% [95% CI: 47.3–58.6]) rats were parasitized by helminth species with a heteroxenous cycle (indirect cycle, with the intervention of a definitive host, and, at least, one intermediate host), but merely 8 rats (2.7% [95% CI: 1.3–5.0]) carried heteroxenous helminth species only. Moreover, 151 rats (50.3% [95% CI: 44.7–56.0] carried monoxenous and heteroxenous helminth species simultaneously.

The species with the highest percentage of parasitation (prevalence) was *H. spumosa*, with 63.7%, and the most abundant was *N. brasiliensis* (13.26). Among zoonotic helminths, *C. hepaticum* was the most prevalent species with 46.3% and *G. neoplasticum* was the most abundant (3.96). The most notable species in terms of the number of helminths of the same species found in an individual rat were *R. nana* with 500 helminths, *N. brasiliensis* with 423 and *G. neoplasticum* with 111 (Table 2).

### 3.2. Helminth Life Cycles

#### 3.2.1. *Hydatigera taeniaeformis* larvae

The larval stage of the zoonotic taenid cestode *H. taeniaeformis* inhabits the liver surface of rats, their intermediate host. The life cycle of this tapeworm [44] is diheteroxenous (Figure S1), with cats and other felines acting as the definitive host carrying the adult stage in the small intestine. Eggs with or from the gravid proglottids are released in the feces of felines which infect rats directly. Eggs develop the larval stage, the strobilocercus in their abdominal cavity, on the liver surface. Finally, felines will be infected after ingestion of an infected rat. The most probable route of infection of humans, although not the only one, seems to be the ingestion of eggs released by felines, which develop into the larval stage.

The prevalence of this tapeworm larva was 1.7%, with only five rats infected, and the presence of one cyst in each one (Table 2).

#### 3.2.2. *Rodentolepis nana*

The adult of the zoonotic hymenolepidid *R. nana*, the dwarf tapeworm, inhabits the small intestine of humans and rats, which act as its only hosts when the cycle [45] is monoxenous (Figure S2), the more common of the two possible kinds of biological cycles of this helminth. The infection of the host occurs after the ingestion of eggs released by another infected host. Then, after emerging from the eggs, the oncospheres develop into cysticercoid-type larval stages in the intestinal mucosa which turn into adult tapeworms in the intestinal lumen. However, much less common, this tapeworm presents a diheteroxenous life cycle, with some arthropods (mainly beetles and fleas) acting as intermediate hosts harboring the cysticercoid. Rats and humans become parasitized after ingesting infected arthropods, accidentally in the case of humans.

The prevalence of this monexenous hymenolepidid was 8.0%, with a median intensity of 29.5 individuals in the parasitized rats (Table 2).

#### 3.2.3. *Hymenolepis diminuta*

The adult of the other zoonotic hymenolepidid tapeworm, *H. diminuta*, the rat tapeworm, also inhabits the small intestine of humans and rats. However, in this case, its life cycle [46] is only diheteroxenous (Figure S3), with some arthropods (also mainly beetles and fleas) acting as intermediate hosts which after ingesting the eggs develop the cysticercoids in their internal cavity. The same as in the case of *R. nana*, rats and humans become parasitized after ingesting infected arthropods, accidentally in the case of humans.

The prevalence of this diheteroxenous hymenolepidid was 21.3%, with a low median intensity of 1.5 individuals only (Table 2).

#### 3.2.4. *Eucoleus gastricus*

The non-zoonotic capillarid nematode *E. gastricus* inhabits the stomach mucosa of rats, causing, sometimes, a visible pathology. Its life cycle [47] seems to be monoxenous (Figure S4). Rats release unembryonated eggs in feces, which under favorable environmental conditions, embryonate in the soil, then being infective to rats. Alternatively, the participation of earthworms acting as paratenic hosts after the ingestion of embryonated eggs and subsequent development of the larval stage seems possible. Rats become infected after ingesting these earthworms.

The prevalence of this monoxenous stomachal capillarid was 29.0%, with a discrete median intensity of 5.1 individuals (Table 2).

#### 3.2.5. *Aonchotheca annulosa*

The other non-zoonotic capillarid nematode *A. annulosa* inhabits the small intestine of rats. Its life cycle [47] is diheteroxenous (Figure S5). Rats release unembryonated eggs in feces, which under favorable environmental conditions, embryonate in the soil. These eggs are ingested by the intermediate host, earthworms, in which the larval stage develops. Rats will be infected after ingesting these earthworms.

The prevalence of this diheteroxenous intestinal capillarid was 4.3% only, but with a non-negligible median intensity of 10.3 individuals (Table 2).

#### 3.2.6. *Calodium hepaticum*

The zoonotic capillarin nematode *C. hepaticum*, also known as *Capillaria hepatica*, typically inhabits the liver parenchyma of rats, but also of humans and other mammals. Its life cycle [47], although being a typically monoxenous cycle (Figure S6), has some particularities. The adults inhabit the liver parenchyma where gravid females lay the unembryonated eggs. As these eggs are trapped in the liver parenchyma, they reach the environment only after the death of their hosts. Then, when a dead infected rat is ingested by a predator or scavenger, the unembryonated eggs pass through the gastrointestinal tract to be released into the environment. Once these unembryonated eggs reach the soil, the embryonation process takes several weeks or months, under favorable conditions. The embryonated eggs are infective to rats or any other mammal, including humans, who accidentally ingest them in contaminated water and food.

The prevalence of this hepatic capillarid was 46.3%, the highest among the zoonotic helminths found (Table 2). However, its median intensity and abundance cannot be recorded due to the limitation of assessing the number of adults of this helminth in each liver. The adults were unrecognizable as individual specimens, with liver parasitism having been detected by the presence of eggs in the liver parenchyma and remains of adult stages and, generally, also by the typical pathology produced by them.

#### 3.2.7. *Trichosomoides crassicauda*

This non-zoonotic trichosomoid nematode inhabits the urinary bladder of rats and has a monoxenous life cycle [36,48] (Figure S7). Males live inside the female reproductive tract. In the urinary bladder the females shed embryonated eggs which are released in urine, in turn, infecting other rats after their ingestion. The eggs hatch in the stomach and the larvae penetrate the gastric wall to initiate, via the portal vein, an intraorganic migration to the urinary bladder, where the adults mature and mate.

The prevalence of this nematode was 6.9%, with a low median intensity of 3.5 individuals (Table 2).

#### 3.2.8. *Nippostrongylus brasiliensis*

The non-zoonotic heligmosomid nematode *N. brasiliensis*, also known as the rat hookworm, inhabits the small intestine of rodents (mainly rats) and presents a monoxenous life cycle [49] (Figure S8). Unembryonated eggs released by females embryonate in the soil, where they hatch and, after two moults, the filariform L_3_ larvae infect other rats transcutaneously, with the oral route of infection also being possible. The L_3_ larvae undergo an intraorganic migration to the lungs and, after ingestion, the young adults reach the small intestine.

The prevalence of the rat hookworm was 36.7%, with the highest median intensity, 36.2 individuals per parasitized rat (Table 2).

#### 3.2.9. *Heterakis spumosa*

The non-zoonotic ascaridid nematode *H. spumosa* inhabits the large intestine of rats. Females release unembryonated eggs, which are shed in rat feces. The eggs, under favorable environmental conditions, embryonate in the soil, being infective to other rats which become infected after their ingestion, thus completing the monoxenous biological cycle [36] (Figure S9).

The prevalence of this monoxenous ascaridid was 63.7%, the highest among all the helminth species found, with a median intensity of 18.6 individuals (Table 2).

#### 3.2.10. *Gongylonema neoplasticum*

The zoonotic spirurid nematode *G. neoplasticum* inhabits the esophagus mucosa of rats, where the female lays embryonated eggs which are released in feces. Its life cycle [50] is diheteroxenous (Figure S10), with some arthropods, mainly cockroaches and beetles, acting as intermediate hosts, which ingest the embryonated eggs. In the hemocoel of the arthropod larval stages develop until the infective L_3_, which encysts in the musculature. Rats and humans become parasitized after ingesting infected arthropods, accidentally in human cases.

The prevalence of this zoonotic spirurid was 36.7%, with a non-negligible median intensity of 10.8 individuals (Table 2).

#### 3.2.11. *Moniliformis moniliformis*

The zoonotic acanthocephalan *M. moniliformis* inhabits the small intestine of rats, where the female lays embryonated eggs which are released in feces. Its life cycle [51] is diheteroxenous (Figure S11), with some arthropods, mainly cockroaches and beetles, acting as intermediate hosts. Once ingested, the acanthor that emerges from the embryonated eggs moults into a second larval stage, the acanthella in the hemocoel of the arthropod, and subsequently turns into an infective cystacanth. Rats and humans become parasitized after ingesting infected arthropods, accidentally in the case of humans.

The prevalence of this zoonotic acanthocephalan was 2.3%, with 7 infected rats, and with a median intensity was 3.7 individuals (Table 2).

### 3.3. Analysis of the Zoonotic Helminth Species

A total of 110 individual rats carried more than 1 zoonotic helminth species (Table 3), including 79 of them carrying 2 zoonotic helminths, 29 with 3 species, and 2 with 4 species. Among these associations, the most frequent is *C. hepaticum* and *G. neoplasticum*. Moreover, the presence of the triple association between *H. diminuta–C. hepaticum–G. neoplasticum* in a total of 17 rats is also worth highlighting. Three of the associations reported are statistically significant: *R. nana*–*M. moniliformis* (χ^2^ = 11.832; *p* = 0.0006); *C. hepaticum*–*G. neoplasticum* (χ^2^ = 36.175; *p* < 0.0001); and *G. neoplasticum*–*M. moniliformis* (χ^2^ = 7.425; *p* = 0.0064).

Prevalences of zoonotic helminth species by host sex and age and by the place and season of capture of hosts are shown in Table 4 and Table 5, respectively. Of note is the higher prevalence observed in adults vs. juveniles for most zoonotic helminth species. However, the differences observed based on sex, place and season of capture are different depending on the helminth species considered.

Adult rats have a higher prevalence of helminth species parasitation than juveniles (odds ratio = 13.4 [95% CI: 6.1–28.3], monoxenous (odds ratio = 7.6 [95% CI: 3.9–14.5] and heteroxenous (odds ratio = 6.8 [95% CI: 3.9–11.8] species. Moreover, the combination of age, sex and the place of capture of the host has a statistically significant influence on the total parasitation of rats by helminths (χ^2^ = 68.857; df = 3; *p* < 0.0001) and the parasitation only by monoxenous helminths (χ^2^ = 52.198; df = 3; *p* < 0.0001), with the highest value being obtained for adult females captured in the sewage system. Likewise, the age and place of capture have a statistically significant influence on the parasitation by heteroxenous helminths (χ^2^ = 63.612; df = 2; *p* < 0.0001), with the highest value being obtained for adults captured in the sewage system.

The binary logistic regression models which best represent the influence that host sex and age, as well as place and season of capture have on the prevalences of the zoonotic helminth species are shown in Table 6. All in all, adult rats have a higher prevalence of zoonotic helminth species parasitation than juveniles (odds ratio = 6.1 [95% CI: 3.6–10.5], and the combination of host age and season of capture has a statistically significant influence on this prevalence. The prevalence of all zoonotic helminth species, with the exception of the acanthocephalan *M. moniliformis*, was statistically influence by at least one of the host factors analyzed.

As shown in Table 5, rats captured in public gardens have a higher prevalence of parasitation by the larva of *H. taeniaeformis* than rats captured in the sewage system (odds ratio = 11.6 [95% CI: 1.9–71.9]). In the case of *R. nana,* rats captured in public gardens also have a higher prevalence of parasitation (odds ratio = 4.6 [95% CI: 1.8–11.8]), and the combination of place and season of host capture (Table 6) has a statistically significant influence on this prevalence, with the highest value being obtained for rats captured in public gardens during the autumn–winter period.

Adult rats have a higher prevalence of parasitation by *H. diminuta* than juveniles of the tapeworm (odds ratio = 2.0 [95% CI: 1.1–3.9]), while for *C. hepaticum* rats captured in the sewage system have a higher prevalence of parasitation by this nematode (Table 5) (odds ratio = 7.5 [95% CI: 4.2–13.3]). Likewise, the combination of place of capture and host age has a statistically significant influence on the *C. hepaticum* prevalence (Table 6), with the highest value being obtained for adults captured in the sewage system.

Finally, adult rats have a higher prevalence of parasitation by *G. neoplasticum* than juveniles (Table 5) (odds ratio = 8.5 [95% CI: 4.2–17.4]), and the combination of place and season of capture as well as host sex has a statistically significant influence on this prevalence (Table 6), with the highest value being obtained for adult males captured in the sewage system during the spring–summer period.

## 4. Discussion

Although the present study has been carried out on 300 specimens of *R. norvegicus* in the city of Barcelona, the fact that only one year period has been analysed should be considered a limitation of the study. Future research on new annual periods may serve to confirm the current results.

Two noteworthy facts make the results presented in this study significant: the results presented are among the few reports of helminths of the urban Norway rat in an important, touristic and sustainable Western European city, as is the case of Barcelona; and the detection of 11 helminth species parasitizing *R. norvegicus*, although they are parasites usually reported in previous studies of this rodent around the world. Moreover, the finding of the six zoonotic helminth species should be highlighted. The helminth species found include: three zoonotic cestodes, the larval stage of a taenid, *H. taeniaeformis*, and the adults of two hymenolepidids *R. nana* and *H. diminuta*; seven nematodes, among them, the zoonotic *C. hepaticum* and *G. neoplasticum*; and the zoonotic acanthocephalan *M. moniliformis*.

In the preliminary study that we previously carried out in that city [30], after the analysis of 100 rat individuals, 5 of the 6 zoonotic helminths had already been detected, with *H. diminuta* being the most prevalent, having a parasitation rate of 33%. However, in the current study, *C. hepaticum* and *G. neoplasticum* are the most prevalent zoonotic helminths. In addition, the larval stage of *H. taeniaeformis* is reported for the first time in Barcelona.

Concerning the zoonotic helminth species only, the analyses of *R. norvegicus* in other European cities [12,13,14,15,16,17,18,28,52,53] revealed the presence of almost the same species, with some specific exceptions. In Spain, the acanthocephalan *M. moniliformis* was reported in Barcelona and also recently in Valencia [28], as well as in several other studies around the world (Malaysia [21]; Peru [22]; Brazil [23]; and South Africa [24]), but not in other European cities. The trematode *Brachylaima* spp. has also been reported in Valencia [28], and previously in Hauts-de-Seine (France) [18] and Palermo (Italy) [15], but so far not in Barcelona. Moreover, the spirurid *Gongylonema* was only found in Palermo (Italy) [15], although reported as *Gongylonema* sp. *Gongylonema neoplasticum* was previously reported in Barcelona [30] and more recently in Valencia [28].

In addition to the studies carried out by our research group in Barcelona and Valencia, *R. nana*, *H. diminuta* and *C. hepaticum* have also been reported in Norway rats in other Spanish locations, such as Granada and Catalonia [25,54]. Likewise, *Trichinella spiralis* (Owen, 1935) has been reported in wild *R. norvegicus* in the Ebro Delta [54].

There is only 1 study carried out 3 decades ago in urban areas of Galicia in which 16 and 19 specimens of *R. norvegicus* were analyzed in the cities of La Coruña and Santiago de Compostela, respectively. However, article only provides information on helminths of the Capillariinae family, highlighting the presence of the zoonotic nematode *C. hepaticum* in the Norway rat [26]. More recently, another study also reported the presence of *C. hepaticum* in a specimen of the Norway rat captured in a peri-urban area of Barcelona [27].

In addition to all the zoonotic helminths mentioned above, a notable absence in the 300 individuals of *R. norvegicus* studied in Barcelona is that of the rat lungworm *A. cantonensis*, already found in Valencia [29] located 300 km south of Barcelona on the Spanish Mediterranean coast.

### 4.1. Zoonotic Helminth Species and Public Health Risk to Humans

Among the six zoonotic species found, *R. nana* is the only one to which urban rats transmit the infective parasitic form, the egg, directly to the human being. In the case of *H. diminuta*, *G. neoplasticum* and *M. moniliformis*, urban rats act as an indirect reservoir (maintaining the biological cycle of the helminth in nature) for humans, who are infected after the accidental ingestion of the intermediate host, an arthropod. Moreover, in the case of *C. hepaticum*, and *H. taeniaeformis*, there are carriers, which are those hosts that directly transmit the parasite to humans; these carriers are the predators of the rat, including, in the case of *C. hepaticum*, other rats.

It is remarkable that a total of 110 of the 206 (53.4%) rats parasitized with zoonotic helminths are carriers of more than 1 of these species. This fact represents an additional risk with regard to potential human infections acquired from *R. norvegicus* in the city of Barcelona. Many of the associations detected are due to two possible factors: these are the zoonotic species with the highest prevalence (*C. hepaticum*, *G. neoplasticum* and *H. diminuta*); they share the same intermediate host (*H. diminuta*, *G. neoplasticum* and *M. moniliformis*, or even *R. nana*).

It is noteworthy that the statistically significant positive association between *G. neoplasticum* and *M. moniliformis* is repeated in six of the seven rats parasitized by this acanthocephalan, underlining the fact that they share the same intermediate host, cockroaches. However, the association between the most prevalent zoonotic species, *C. hepaticum*, and *M. moniliformis* is only repeated on two occasions. On the other hand, the positive association, also statistically significant, between the 2 most prevalent species seems to be due to the high number of rats parasitized by both helminths, which coincide on 76 occasions, taking into account both the associations of 2 or more helminth species.

#### 4.1.1. *Hydatigera taeniaeformis* larvae

Rats play an indirect role in the transmission of this tapeworm to humans, acting as reservoirs of the helminth but not transmitting it directly to humans (Section 3.2.1).

As already mentioned, humans become infected with this zoonotic tapeworm after the accidental ingestion of the eggs released by felines which contaminate water, vegetables and other food. Cases of strobilocercus larvae in humans are rare [55,56], although there are several published reports, genuine or spurious, of human parasitation with the adult tapeworm in Argentina, Japan and Sri Lanka [57].

The higher prevalence of this larval stage in rats captured in public gardens than in in the sewage system might be related to the presence of infected cats, not in sewers, but on the ground in the city. Cats release the eggs in their feces, particularly in sandy soil and/or grass, making infestation of rats in these environments more likely. This fact also puts humans at risk, mainly workers and users of public parks and gardens, and therefore, preventive measures ought to be taken to diminish the risk of parasitation.

#### 4.1.2. *Rodentolepis nana*

The direct life cycle of this zoonotic tapeworm is more frequent than the indirect life cycle (Section 3.2.2). Therefore, rats play a significant role in the infection of humans as the tapeworm eggs, directly infective to humans, are released in rat feces, contaminating water, vegetables, dirty hands, etc. In addition, rats act at the same time as reservoirs of this helminth in the less frequent indirect life cycle, i.e., with the participation of beetles and other insects as intermediate hosts.

The dwarf tapeworm is a neglected zoonotic parasite affecting more than 75 million people worldwide, mainly in Asia, Central and South America, Africa and Southern and Eastern Europe [58,59]. Although this hymenolepiasis is usually asymptomatic in humans, the parasitation can sometimes cause abdominal pain, diarrhea and anorexia, among other symptoms [60].

The highest prevalence of this tapeworm was found in rats captured in public gardens, in which humans could become parasitized by the infective eggs released in rat feces. Therefore, as in the case of *H. taeniaeformis* larvae, it is necessary to take preventive measures to diminish the risk of parasitation.

#### 4.1.3. *Hymenolepis diminuta*

*Hymenolepis diminuta* has an indirect life cycle, with rats acting as reservoirs of this tapeworm. Therefore, humans can only become infected after the ingestion of infected intermediate hosts (Section 3.2.3).

This diheteroxenous tapeworm is more prevalent in adult rats than in juveniles, probably related to the diet of the rats, i.e., in older rats the diet presumably includes a greater number of arthropods [31], and, in turn, older rats have had a greater risk of becoming infected than juveniles.

Cases of human parasitism by *H. diminuta* are rare due to the route of infection. In fact, in the last 2 centuries, only approximately 1600 human cases have been reported worldwide, most of them in the Americas, Southeast Asia and the eastern Mediterranean [61]. Cases have also been reported in various European countries, such as Spain [62], Italy [63,64], Poland [65] and Romania [66]. Symptomatic infections in humans can mainly cause intestinal symptoms, such as abdominal pain, diarrhea, fever and anorexia [61].

Although human infestations appear to be incidental, given that the rat tapeworm prevalence is >20%, users of public gardens, especially children, should be made aware of the risk of the accidental ingestion of arthropods, intermediate hosts of *H. diminuta* and other zoonotic helminths.

#### 4.1.4. *Calodium hepaticum*

The hepatic nematode *C. hepaticum* is a common parasite of rats. However, due to its low specificity, other mammals, including humans, can also become infected [67]. Parasitized rats play a reservoir role (Section 3.2.6).

Human infections (due to the accidental ingestion of infective eggs contaminating water and food) are rather rare, with approximately 200 cases worldwide. The most common symptoms include hepatomegaly, leukocytosis with eosinophilia and fever, being fatal without adequate and early diagnosis in more than 50% of cases [67].

The highest prevalences of this monoxenous nematode found in our study were in adult rats captured during the spring–summer period. Older rats have had a greater risk of becoming infected than juveniles which could be related to the higher mobility of older rat individuals, increasing the exposure time and in turn the likelihood of being infected, in this case, ingesting an infective egg. Moreover, the eggs embryonate in the soil to become infective, a development that is presumably more effective in the spring–summer period than in colder months. Therefore, the increased presence in the environment of infective eggs during the spring–summer period must be considered to minimize the risk to humans, mainly concerning sewage system workers as well as users and workers of public parks and gardens.

#### 4.1.5. *Gongylonema neoplasticum*

In the case of this esophageal nematode, and as in the case of *H. diminuta,* humans become infected by accidentally ingesting the arthropod intermediate hosts, mainly cockroaches in the case of *G. neoplasticum*, (Section 3.2.10).

The highest prevalence of *G. neoplasticum* was found in adult male rats captured in the sewage system in the spring–summer period. This is presumably related to the diet of the older rats, the higher mobility of males, and the higher population of the main intermediate host, cockroaches, in the sewage system during the spring–summer period.

More than 50 human cases of *Gongylonema* sp. infections, mainly in the esophagus or the oral cavity, have been reported worldwide, including European countries, such as Italy, the former Soviet Union, Bulgaria, Austria, Germany and Hungary [68,69], and more recently Spain [70], France [71,72], Slovenia [73] and again Bulgaria [74]. The most common symptoms in humans can be strange sensations in the oral cavity and/or symptoms related to reflux, indicating a possible esophageal condition [69].

Although human cases have been reported as caused by *Gongylonema pulchrum* Molin, 1857, this species presents a wide spectrum of definitive hosts, mainly domestic and wild pigs, while *G. neoplasticum* is an exclusive parasite of murids of the genus *Rattus*, mainly *R. norvegicus* and *R. rattus*. This reason could explain the low incidence of *G. pulchrum* in urban areas, with *G. neoplasticum*, probably being the predominant zoonotic species in urban areas where its definitive hosts, synanthropic rodents, are found [75].

As in the case of *H. diminuta*, the risk of human infestation appears to be rare, but the prevalence of *G. neoplasticum* observed in the rats studied is close to 37% (Table 5). Therefore, clinicians should consider gongylonemiasis in the differential diagnosis of clinical cases in which the patient experiences the above-mentioned symptoms, particularly in those cities where the parasite has already been found.

#### 4.1.6. *Moniliformis moniliformis*

As in the cases of *H. diminuta* and *G. neoplasticum*, this zoonotic acanthocephalan has an indirect life cycle, and consequently rats act only as reservoirs of this helminth, with humans being infected when they accidentally ingest infected cockroaches, the main intermediate host of *M. moniliformis*.

Among the zoonotic helminths reported with an indirect life cycle, *M. moniliformis* has the lowest prevalence of parasitation in the rats in Barcelona, which could explain the absence of statistically significant influences of the factors analyzed on its prevalence. However, as expected, the highest prevalence corresponds to adult male rats living in the sewage system, which is consistent with the fact, above-mentioned for *G. neoplasticum*, that its main intermediate host is the cockroach.

Human cases of this intestinal acanthocephalan are rare and have occurred in several countries around the world, mostly in children, presenting with non-specific gastrointestinal symptoms such as diarrhea, abdominal pain and vomiting [76,77]. Therefore, as in the case of *G. neoplasticum*, health professionals, mainly pediatricians, should consider human acanthocephaliasis in the differential diagnosis if the patient experiences the above-mentioned symptoms.

## 5. Conclusions

The results obtained show that urban rats are carriers of zoonotic helminth parasites (at least five species in the case of Barcelona), for which rats can act, depending on the helminth species, transmitting the infective parasitic form directly to the human being, or acting as an indirect reservoir.

As rodents are present in all urban areas, the entire society can be exposed to these parasitic zoonoses. However, due to their proximity to the most important transmission sites, the groups most at risk of acquiring rat-borne helminth zoonosis are maintenance workers of the sewage system, parks and gardens, wastewater treatment and disposal units. Furthermore, visitors to green spaces, especially children and elderly people, should also be considered population groups at risk.

Consequently, health services as well as occupational risk prevention services should be aware of zoonotic helminths transmitted by urban rats in their city in order to provide, from a One Health approach, adequate information and implement preventive measures to protect the general public and the workers involved in particular.

## Figures and Tables

**Table 1:** Selected characteristics of the 300 *Rattus norvegicus* analyzed.

Host Sex *	Host Age *	Season of Capture				
Males		Autumn	Winter	Spring	Summer	TOTAL
	Juveniles	1	33	--	11	45
	Adults	15	58	19	16	108
	TOTAL	16	91	19	27	153
Females		Autumn	Winter	Spring	Summer	TOTAL
	Juveniles	3	37	3	9	52
	Adults	7	48	13	20	88
	TOTAL	10	85	16	29	140

* In a total of 7 rat individuals, neither sex nor age could be determined.

**Table 2:** Selected characteristics of the helminth community of 300 *Rattus norvegicus* analyzed.

Helminth Species	Site	LC	Zoonotic	n	Prevalence (%) (95% CI)	Mean Abundance (SE)	Median Intensity (Range)
**CESTODA**							
*Taenia taeniaeformis* larvae	liver	DH	yes	5	1.7 (0.6–3.6)	0.02 (0.01)	1 (1)
*Rodentolepis nana*	small intestine	MX/DH	yes	24	8.0 (5.3–11.5)	2.36 (1.70)	29.5 (1–500)
*Hymenolepis diminuta*	small intestine	DH	yes	64	21.3 (17.0–26.2)	0.33 (0.05)	1.5 (1–12)
**NEMATODA**							
*Eucoleus gastricus*	stomach	MX	no	87	29.0 (24.1–34.3)	1.48 (0.19)	5.1 (1–25)
*Aonchotheca annulosa*	small intestine	DH	no	13	4.3 (2.5–7.1)	0.44 (0.18)	10.2 (1–32)
*Calodium hepaticum* *	liver	MH	yes	139	46.3 (40.7–52.0)	---	---
*Trichosomoides crassicauda* **	urinary bladder	MH	no	10	6.6 (3.4–11.4)	0.23 (0.09)	3.5 (1–10)
*Nippostrongylus brasiliensis*	small intestine	MH	no	110	36.7 (31.4–42.2)	13.26 (2.84)	36.2 (1–423)
*Heterakis spumosa*	large intestine	MH	no	191	63.7 (58.1–69.0)	11.87 (1.06)	18.6 (1–91)
*Gongylonema neoplasticum*	esophagus	DH	yes	110	36.7 (31.4–42.2)	3.96 (0.67)	10.8 (1–111)
**ACANTHOCEPHALA**							
*Moniliformis moniliformis*	small intestine	DH	yes	7	2.3 (1.0–4.5)	0.08 (0.04)	3.6 (1–9)

LC—life cycle, DH—diheteroxenous life cycle, MX—monoxenous life cycle, n—number of infested hosts, CI—confidence interval, and SE—standard error. * In the case of *Calodium hepaticum*, quantitative data were not recorded as it was impossible to determine the number of adults of this helminth in each liver. ** In the case of *Trichosomoides crassicauda*, the number of urinary bladders analyzed was 152 only; the results for this helminth take this fact into account.

**Table 3:** Co-infections of two, three or four zoonotic helminth species detected in the analyzed rats.

Zoonotic Helminth Species	Number of Rats
*H. taeniaeformis–H. diminuta*	1
*H. taeniaeformis–C. hepaticum*	1
*R. nana–H. diminuta*	1
*R. nana–C. hepaticum*	5
*R. nana–G. neoplasticum*	2
*H. diminuta–C. hepaticum*	14
*H. diminuta–G. neoplasticum*	3
*C. hepaticum–G. neoplasticum*	52
*H. taeniaeformis–R. nana–C. hepaticum*	1
*R. nana–H. diminuta–C. hepaticum*	1
*R. nana–H. diminuta–G. neoplasticum*	1
*R. nana–C. hepaticum–G. neoplasticum*	4
*R. nana–G. neoplasticum–M. moniliformis*	2
*H. diminuta–C. hepaticum–G. neoplasticum*	17
*H. diminuta–G. neoplasticum–M. moniliformis*	2
*C. hepaticum–G. neoplasticum–M. moniliformis*	1
*H. taeniaeformis–H. diminuta–C. hepaticum–G. neoplasticum*	1
*R. nana–C. hepaticum–G. neoplasticum–M. moniliformis*	1

**Table 4:** Prevalence of parasitation of the zoonotic helminth species found in *Rattus norvegicus* by host sex and age.

		*Hydatigera taeniaeformis* l.	*Rodentolepis nana*	*Hymenolepis diminuta*	*Calodium hepaticum*	*Gongylonema neoplasticum*	*Moniliformis moniliformis*	Total of Zoonotic Helminth Species
**Place of capture**								
Sewage system	n (prevalence)	2 (0.8%)	16 (6.1%)	55 (20.9%)	126 (47.9%)	106 (40.3%)	7 (2.7%)	182 (69.2%)
	95% CI	0.2–2.4	3.7–9.5	16.3–26.1	41.9–53.9	34.5–46.3	1.2–5.2	63.4–74.5
Public gardens	n (prevalence)	3 (8.1%)	8 (21.6%)	9 (24.3%)	13 (35.1%)	4 (10.8%)	---	24 (64.9%)
	95% CI	2.3–20.1	10.8–36.7	12.8–39.7	21.3–51.2	3.8–23.7	---	48.8–78.7
**Season of capture**								
Autumn–Winter	n (prevalence)	5 (2.4%)	23 (11.0%)	43 (20.6%)	69 (33.0%)	58 (27.8%)	6 (2.9%)	130 (62.2%)
	95% CI	0.9–5.2	7.3–15.8	15.5–26.4	26.9–39.6	22.0–34.1	1.2–5.8	55.5–68.6
Spring–Summer	n (prevalence)	---	1 (1.1%)	21 (23.1%)	70 (76.9%)	52 (57.1%)	1 (1.1%)	76 (83.5%)
	95% CI	---	0.1–5.0	15.3–32.5	67.5–84.7	46.9–67.0	0.1–5.0	74.9–90.0

**Table 5:** Prevalence of parasitation of the zoonotic helminth species found in *Rattus norvegicus* by place and season of capture.

		*Hydatigera taeniaeformis* l.	*Rodentolepis nana*	*Hymenolepis diminuta*	*Calodium hepaticum*	*Gongylonema neoplasticum*	*Moniliformis moniliformis*	Total of Zoonotic Helminth Species
**Place of capture**								
Sewage system	n (prevalence)	2 (0.8%)	16 (6.1%)	55 (20.9%)	126 (47.9%)	106 (40.3%)	7 (2.7%)	182 (69.2%)
	95% CI	0.2–2.4	3.7–9.5	16.3–26.1	41.9–53.9	34.5–46.3	1.2–5.2	63.4–74.5
Public gardens	n (prevalence)	3 (8.1%)	8 (21.6%)	9 (24.3%)	13 (35.1%)	4 (10.8%)	---	24 (64.9%)
	95% CI	2.3–20.1	10.8–36.7	12.8–39.7	21.3–51.2	3.8–23.7	---	48.8–78.7
**Season of capture**								
Autumn–Winter	n (prevalence)	5 (2.4%)	23 (11.0%)	43 (20.6%)	69 (33.0%)	58 (27.8%)	6 (2.9%)	130 (62.2%)
	95% CI	0.9–5.2	7.3–15.8	15.5–26.4	26.9–39.6	22.0–34.1	1.2–5.8	55.5–68.6
Spring–Summer	n (prevalence)	---	1 (1.1%)	21 (23.1%)	70 (76.9%)	52 (57.1%)	1 (1.1%)	76 (83.5%)
	95% CI	---	0.1–5.0	15.3–32.5	67.5–84.7	46.9–67.0	0.1–5.0	74.9–90.0

**Table 6:** Binary logistic regression models for the prevalences of the zoonotic helminth species of *Rattus norvegicus* by host sex and age, place and season of capture (*) by *χ*^2^ values with associated probabilities (*p)* for the model created including independent variables. Only statistically significant models are reported.

Zoonotic Helminth Species/Independent Variables	df	*χ* ^2^	*p*
*Hydatigera taeniaeformis larvae*			
Place of capture	1	6.561	0.010
*Rodentolepis nana*			
Place of capture	1	8.724	0.03
Place and season of capture	2	16.928	<0.0001
*Hymenolepis diminuta*			
Host age	1	4.921	0.027
*Calodium hepaticum*			
Season of capture	1	55.876	<0.0001
Season of capture and host age	2	62.754	<0.0001
*Gongylonema neoplasticum*			
Host age	1	48.551	<0.0001
Host age and place of capture	2	72.525	<0.0001
Host age and place and season of capture	3	84.877	<0.0001
Host age and sex and place and season of capture	4	89.227	<0.0001
Total of helminth zoonotic species			
Host age	1	46.329	<0.0001
Host age and season of capture	2	58.624	<0.0001

df—degree of freedom. * Season of capture was grouped in two periods, autumn–winter and spring–summer, due to the small number of rats in autumn and spring.

## Data Availability

The data presented in this study are available on request from the corresponding author.

## Supplementary Materials

The following supporting information can be downloaded at: www.mdpi.com/article/10.3390/ani15030298/s1, Figure S1. Life cycle of *Hydatigera taeniaeformis* (illustration by A. L. Debenedetti); Figure S2. Life cycle of *Rodentolepis nana* (illustration by A. L. Debenedetti); Figure S3. Life cycle of *Hymenolepis diminuta* (illustration by A. L. Debenedetti); Figure S4. Life cycle of *Eucoleus gastricus* (illustration by A. L. Debenedetti); Figure S5. Life cycle of *Aonchotheca annulosa* (illustration by A. L. Debenedetti); Figure S6. Life cycle of *Calodium hepaticum* (illustration by A. L. Debenedetti); Figure S7. Life cycle of *Trichosomoides crassicauda* (illustration by A. L. Debenedetti); Figure S8. Life cycle of *Nippostrongylus brasiliensis* (illustration by A. L. Debenedetti); Figure S9. Life cycle of *Heterakis spumosa* (illustration by A. L. Debenedetti); Figure S10. Life cycle of *Gongylonema neoplasticum* (illustration by A. L. Debenedetti); Figure S11. Life cycle of *Moniliformis moniliformis* (illustration by A. L. Debenedetti).
