# Supplementary material for: The Role of the Norway Rat, Rattus norvegicus, as a Reservoir of Zoonotic Helminth Species in the City of Barcelona (Spain)
Source: Animals (Basel). 2025 Jan 21;15(3):298. doi: 10.3390/ani15030298 (PMC11816058; doi:10.3390/ani15030298)
Supplement: Supplementary file 1 [file animals-15-00298-s001.zip › Figure captions - Figure 1 to Figure 11.pdf]

Figure captions: Figure 1 to Figure 11

Figure S1.- Life cycle of *Hydatigera taeniaeformis* (illustration by A. L. Debenedetti).

Figure S2.- Life cycle of *Rodentolepis nana* (illustration by A. L. Debenedetti).

Figure S3.- Life cycle of *Hymenolepis diminuta* (illustration by A. L. Debenedetti).

Figure S4.- Life cycle of *Eucoleus gastricus* (illustration by A. L. Debenedetti).

Figure S5.- Life cycle of *Aonchotheca annulosa* (illustration by A. L. Debenedetti).

Figure S6.- Life cycle of *Calodium hepaticum* (illustration by A. L. Debenedetti).

Figure S7.- Life cycle of *Trichosomoides crassicauda* (illustration by A. L. Debenedetti).

Figure S8.- Life cycle of *Nippostrongylus brasiliensis* (illustration by A. L. Debenedetti).

Figure S9.- Life cycle of *Heterakis spumosa* (illustration by A. L. Debenedetti).

Figure S10.- Life cycle of *Gongylonema neoplasticum* (illustration by A. L. Debenedetti).

Figure S11.- Life cycle of *Moniliformis moniliformis* (illustration by A. L. Debenedetti).
