# Supplementary figures and images for: The Role of the Norway Rat, Rattus norvegicus, as a Reservoir of Zoonotic Helminth Species in the City of Barcelona (Spain)
Source: Animals (Basel). 2025 Jan 21;15(3):298. doi: 10.3390/ani15030298 (PMC11816058; doi:10.3390/ani15030298)

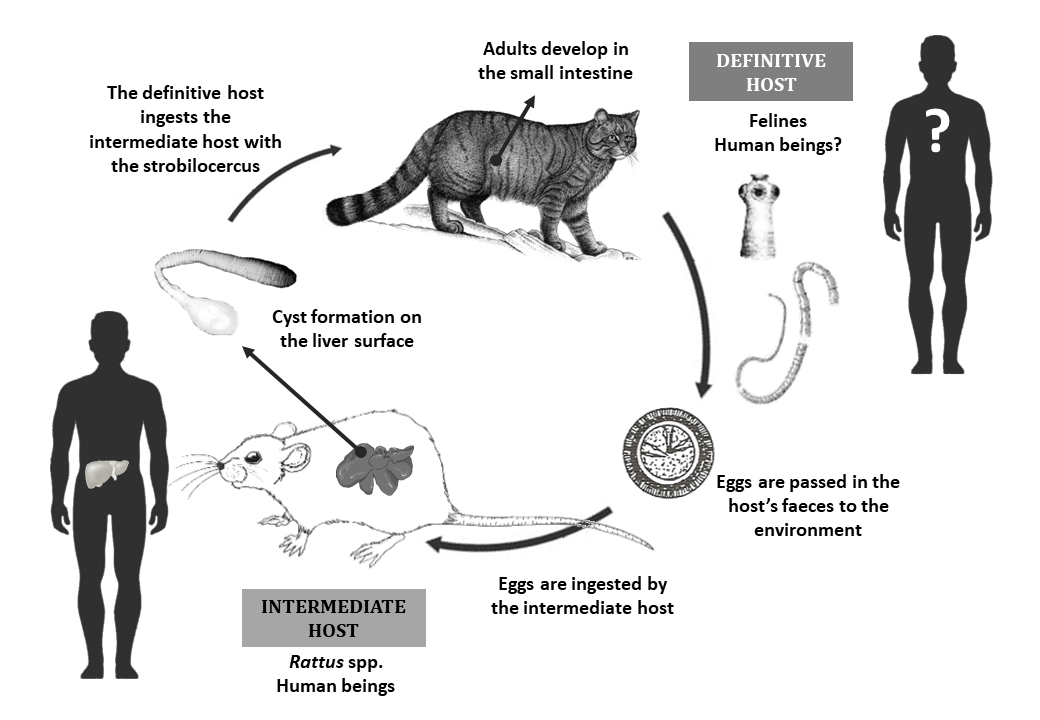

Supplement: Supplementary file 1 [file animals-15-00298-s001.zip › Figure S1.- Hydatigera taeniaeformis.png]

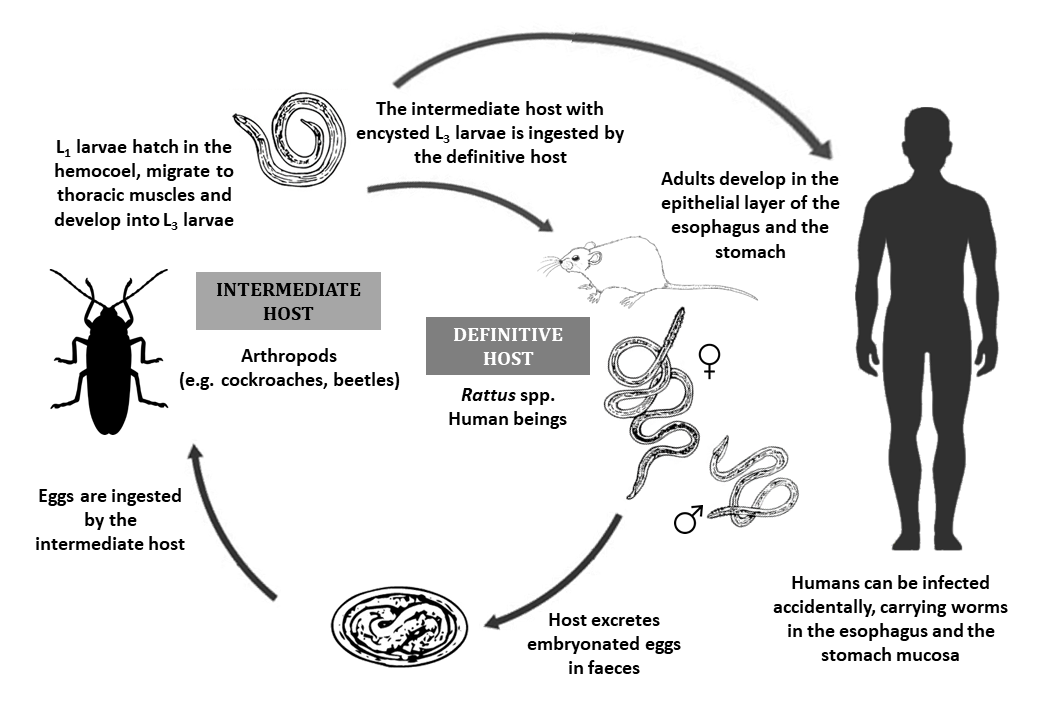

Supplement: Supplementary file 1 [file animals-15-00298-s001.zip › Figure S10.- Gongylonema neoplasticum.PNG]

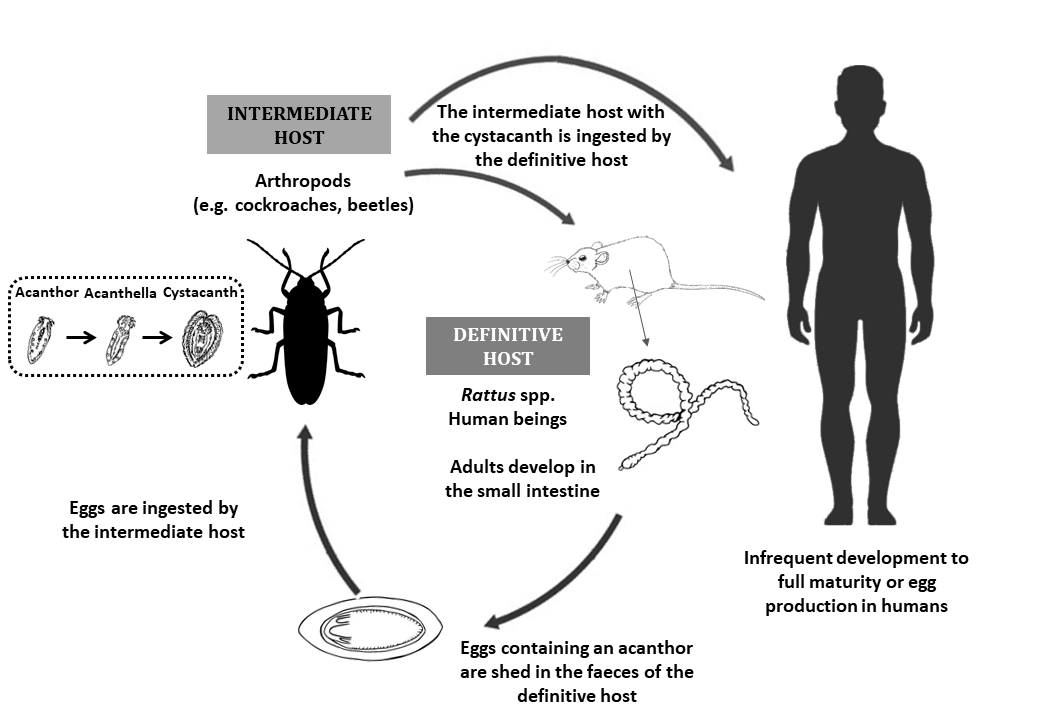

Supplement: Supplementary file 1 [file animals-15-00298-s001.zip › Figure S11.- Moniliformis moniliformis.PNG]

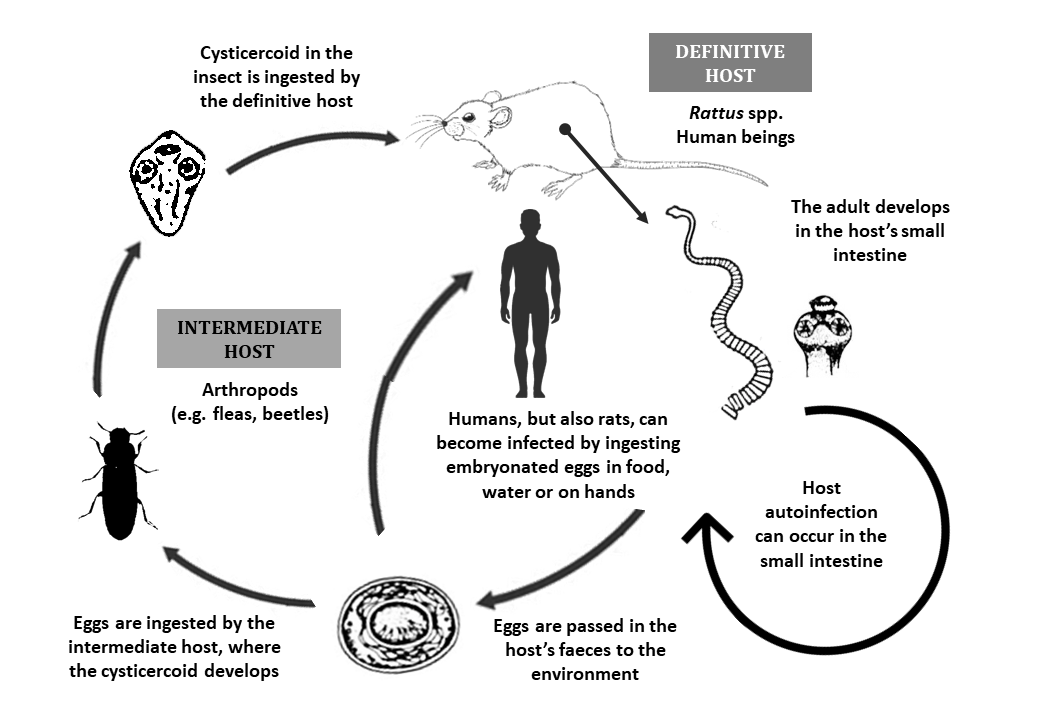

Supplement: Supplementary file 1 [file animals-15-00298-s001.zip › Figure S2.- Hymenolepis nana.PNG]

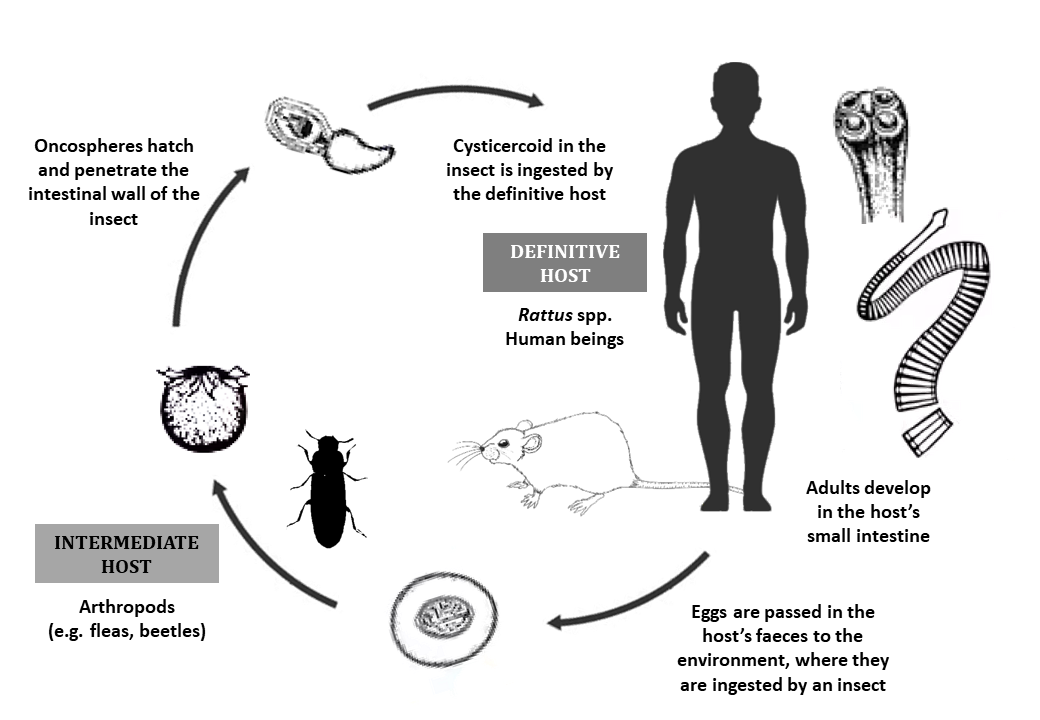

Supplement: Supplementary file 1 [file animals-15-00298-s001.zip › Figure S3.- Hymenolepis diminuta.png]

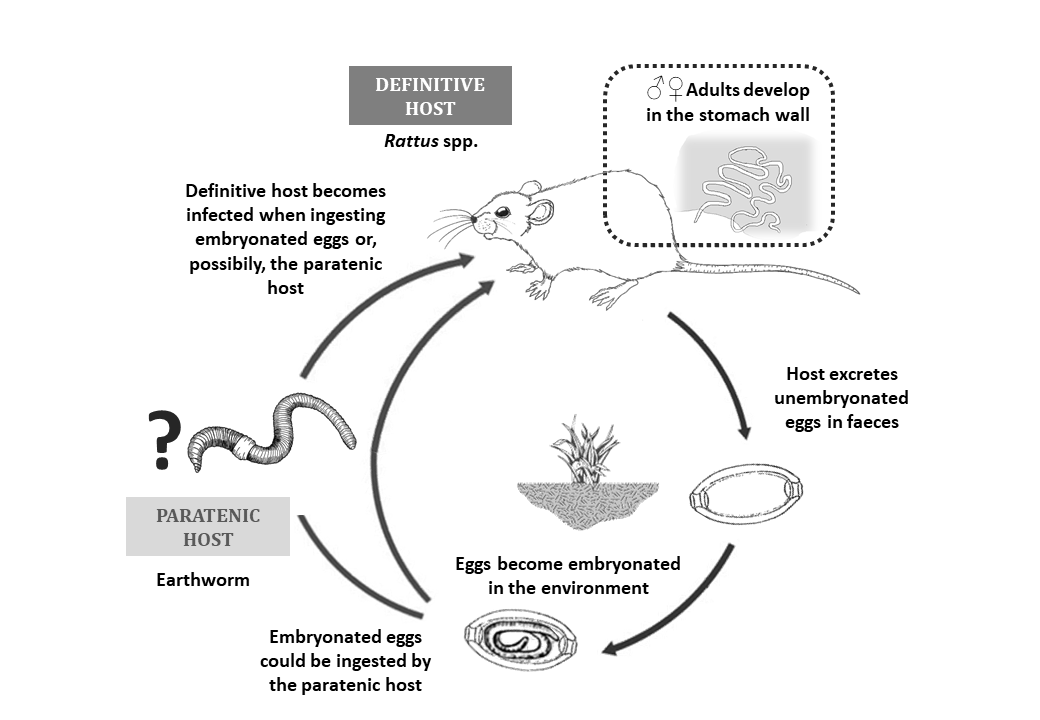

Supplement: Supplementary file 1 [file animals-15-00298-s001.zip › Figure S4.- Eucoleus gastricus.png]

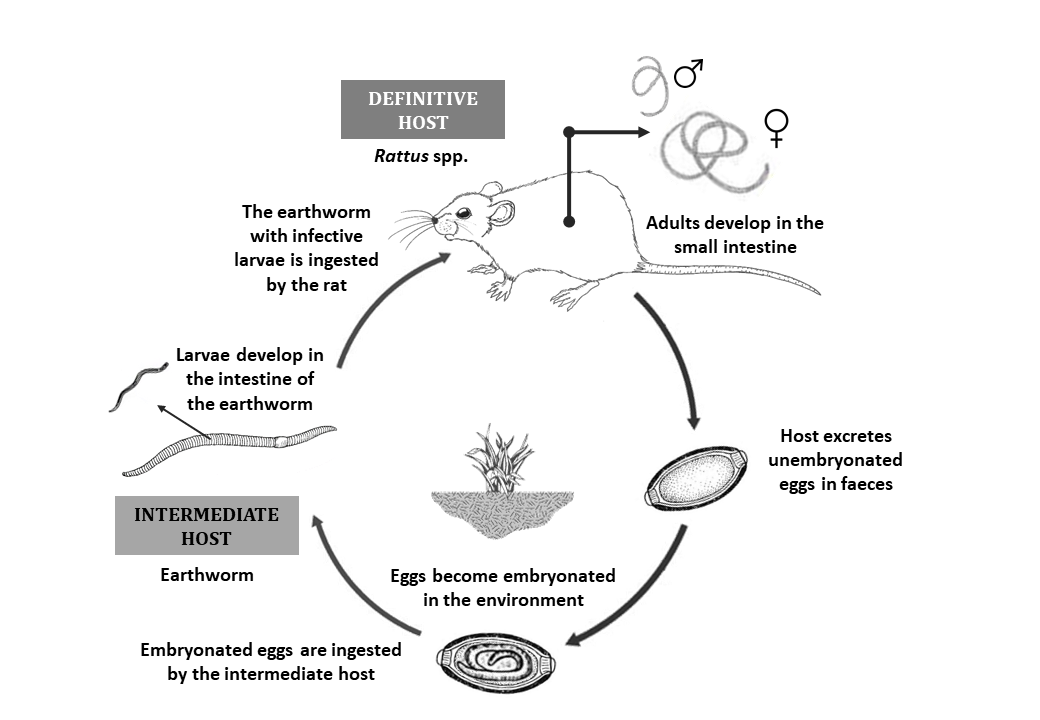

Supplement: Supplementary file 1 [file animals-15-00298-s001.zip › Figure S5.- Aonchotheca annulosa.PNG]

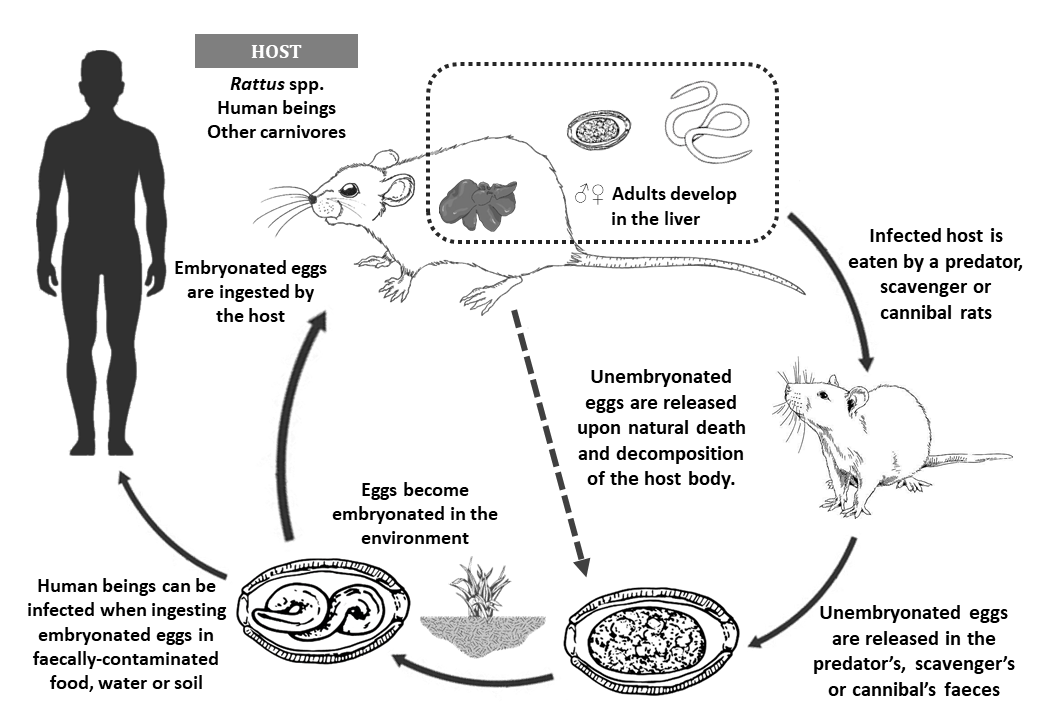

Supplement: Supplementary file 1 [file animals-15-00298-s001.zip › Figure S6.- Calodium hepaticum.PNG]

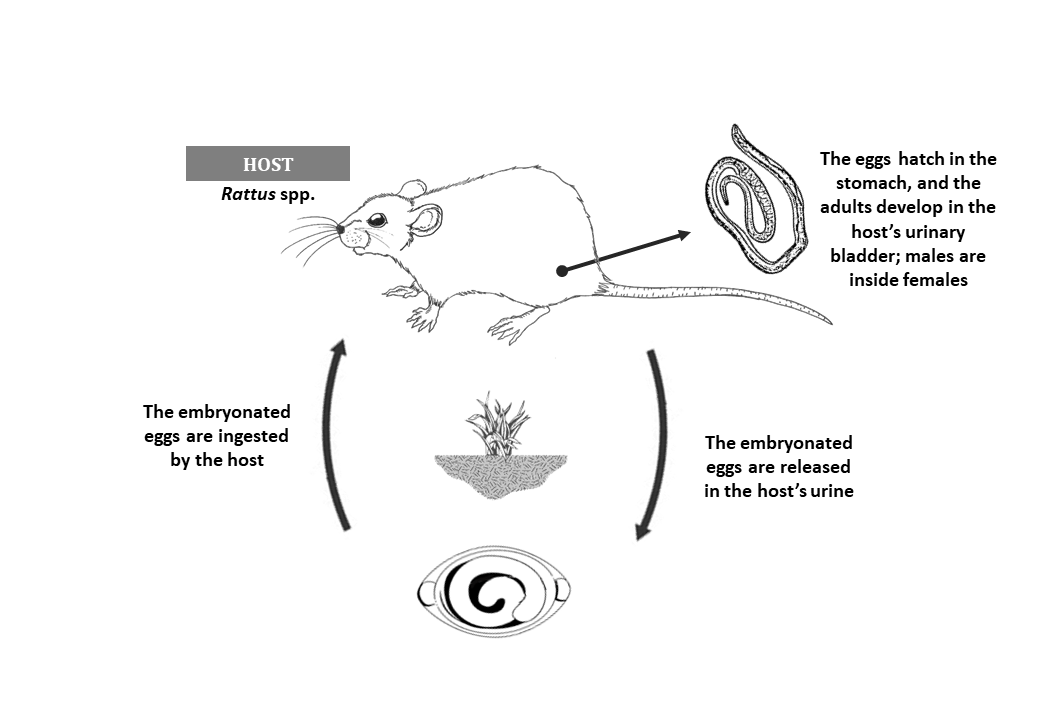

Supplement: Supplementary file 1 [file animals-15-00298-s001.zip › Figure S7.- Trichosomoides crassicauda.png]

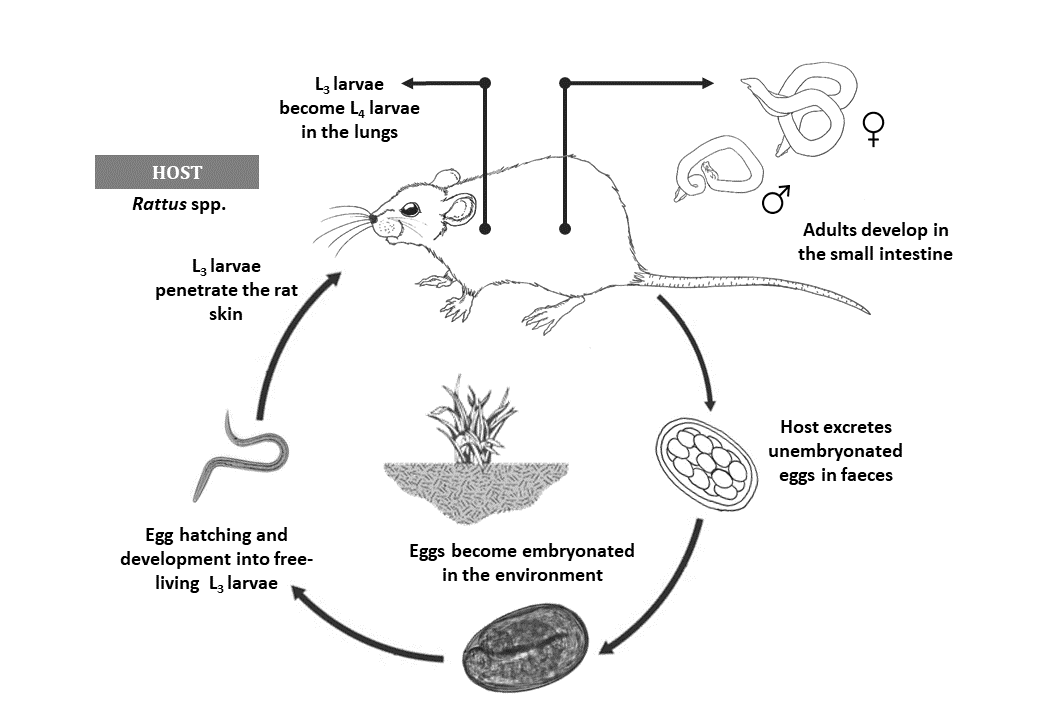

Supplement: Supplementary file 1 [file animals-15-00298-s001.zip › Figure S8.- Nippostrongyluis brasiliensis.PNG]

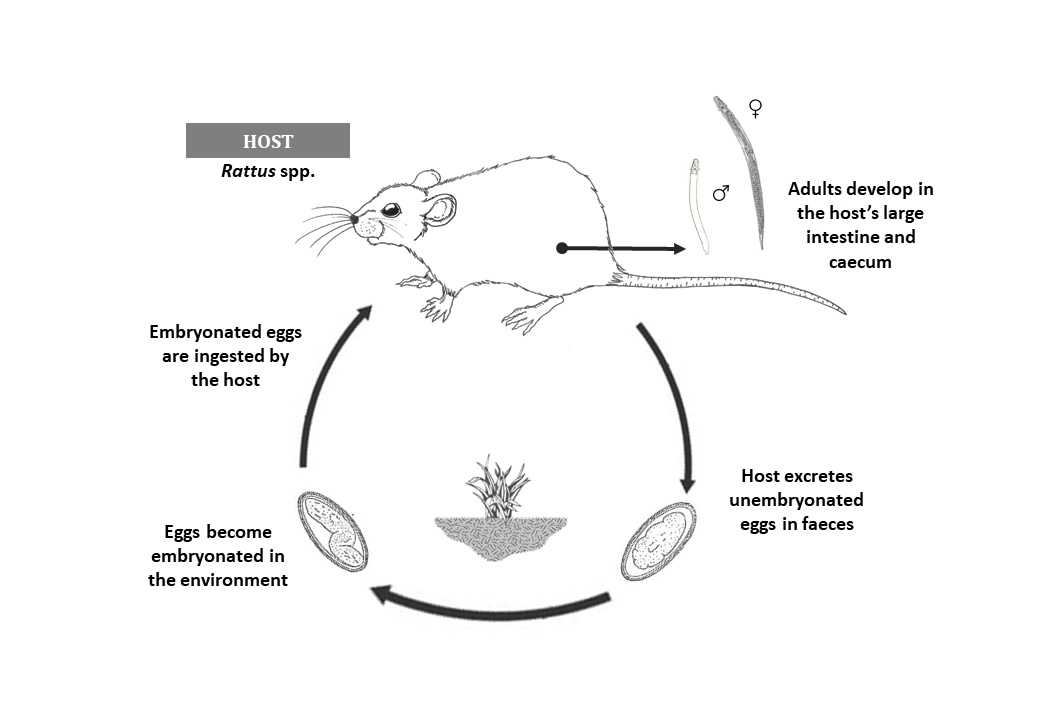

Supplement: Supplementary file 1 [file animals-15-00298-s001.zip › Figure S9.- Heterakis spumosa.PNG]
